# Critical issues in experimental studies of prosociality in non-human species

**DOI:** 10.1007/s10071-016-0973-6

**Published:** 2016-03-21

**Authors:** S. Marshall-Pescini, R. Dale, M. Quervel-Chaumette, F. Range

**Affiliations:** Comparative Cognition, Messerli Research Institute, University of Veterinary Medicine, Vienna, Medical University of Vienna, University of Vienna, Veterinärplatz 1, 1210 Vienna, Austria; Wolf Science Centre, Ernstbrunn, Austria

**Keywords:** Prosociality, Helping, Altruism, Comparitive cognition

## Abstract

Prosociality and acts of altruism are defined as behaviours which benefit another with either no gain or some immediate cost to the self. To understand the evolutionary origins of these behaviours, in recent years, studies have extended to primate species; however, studies on non-primates are still scarce. In light of the fact that phylogenetic closeness to humans does not appear to correlate with prosocial tendencies, but rather differences in the propensity towards prosociality may be linked to allomaternal care or collaborative foraging, it appears that convergent selection pressures may be at work in the evolution of prosociality. It would hence seem particularly important to extend such studies to species outside the primate clade, to allow for comparative hypothesis testing of the factors affecting the evolution of prosocial behaviours. In the current review, we focus on the experimental paradigms which have been used so far (i.e. the prosocial choice task, helping paradigms and food-sharing tests) and highlight the strengths and weaknesses of each method. In line with the aim of encouraging a broader comparative approach to the topic of prosociality, particular emphasis is placed on the methodological issues that need to be taken into account. We conclude that although a number of the paradigms used so far may be successfully applied to non-primate species, there is a need to simplify the cognitive demands of the tasks and ensure task comprehension to allow for a ‘fair’ comparative approach of prosocial tendencies across species.

## Introduction

A prosocial behaviour is usually defined as a voluntary behaviour that benefits another (Jensen et al. 2014). Typically, the distinction between an altruistic and a prosocial behaviour has been based on the fact that whereas prosociality need not involve an immediate cost to the actor, altruism necessarily does (Silk 2007). For instance ‘helping’ behaviours, which imply an immediate cost to the actor, would be considered not just prosocial (in that the action benefits another) but also altruistic, because they are costly to perform. Both these terms are generally distinguished from the term ‘cooperation’, since the latter implies a joint, synchronized (potentially complementary) action performed by two (or more) individuals (Brosnan and de Waal 2002; Boesch and Boesch 1989), whereas both in prosociality and altruism only the actor is involved in a behaviour that results in a benefit for the partner.

Most authors describe prosocial and altruistic acts purely in terms of the cost and benefits to the actor and the receiver rather than involving a ‘motivational’ element underlying such actions. However, a number of authors do include a concern for others as the motivational drive to act prosocially in their definitions (i.e. ‘other-regarding preferences’), and consider this to be the crucial element defining such behaviours (de Waal 2008; Burkart et al. 2007).

Another important consideration when navigating the literature on prosociality and altruism is that the same terminology may be used differently depending on the research field, whether the study involves laboratory or fieldwork, and whether an ultimate or proximate level of explanation is being referred to (see Scott-Phillips et al. 2011; de Waal 2008; West et al. 2007 for an insightful discussion on this topic). For example, evolutionary biologists refer to ‘cooperation’ and ‘altruism’ in terms of the net effects (costs and benefits) on direct fitness for the actor and the receiver. However, psychologists may use the same ‘cost and benefit’ terms to refer to the immediate costs to the actor (such as in ‘economic games paradigms’), which do not necessarily reflect the benefits at the fitness level. Furthermore, their definitions typically include a reference to the underlying psychological motivation (e.g. ‘other-regarding preferences’) of such actions and/or the underlying cognitive mechanism/s (e.g. perspective taking, theory of mind). The latter approach hence focuses on the proximate rather than the ultimate level of explanation (Scott-Phillips et al. 2011; de Waal 2008; West et al. 2007).

Different hypotheses have been put forward in terms of both the ultimate and proximate mechanisms driving prosociality. In terms of the ultimate level of explanation, a number of mechanisms have been recognized as having the capacity to maintain prosocial behaviours within a population. Kin selection can facilitate prosocial behaviours between genetically related individuals through indirect fitness benefits gained by the actor who performs the prosocial behaviour (Hamilton 1964; Axelrod and Hamilton 1981). Reciprocity (where prosocial behaviours are conditional upon having received similar behaviours from others) can maintain such altruistic-like behaviours among unrelated individuals, if ultimately such acts increase the actor’s inclusive fitness (Trivers 1971; Brosnan and Bshary 2010; Lehmann and Rousset 2010; see also West et al. 2007 for a review).

Similarly, at the proximate level, different mechanisms have been suggested to underlie prosocial behaviours. Empathy, i.e. sharing the same emotion observed in another, and sympathy, i.e. the ability to feel concern for others, are thought to be the main mechanisms leading to prosocial behaviours in humans (Batson et al. 1981; Eisenberg and Miller 1987). Indeed a number of authors have suggested that the same processes underlie prosocial behaviours in non-human species as well (Preston and de Waal 2002; de Waal 2008), with these prosocial motivations potentially being mediated by oxytocin (Madden and Clutton-Brock 2011). However, other authors have argued that prosocial behaviours are simply a product of a high motivation for sociality (or social tolerance: Yamamoto et al. 2009), or a strategy adopted to avoid harassment from conspecifics (Stevens 2004; Gilby 2006). For still other authors, proactive prosociality needs to be supported by sophisticated cognitive capacities such as cultural learning, theory of mind, perspective taking and moral judgement which are thought to be present only in humans (e.g. Silk et al. 2005).

In contrast to the vast literature on cooperation, which mainly focuses on the ultimate levels of explanation, researchers investigating prosociality have focused more on tracing the origin of *human* prosociality, attempting to discern whether any prosocial behaviours occur in our closest living relatives and what may be the underlying mechanism—both emotional and cognitive—of its expression. In line with this objective, experimental studies have mostly concentrated on primates (as will emerge strongly from the present review). However, intriguingly, results have shown no clear link between phylogenetic closeness to humans and higher prosocial tendencies (reviewed in Cronin 2012; Jaeggi et al. 2010a; Silk and House 2011) which has been taken to suggest that other convergent selection pressures may be at work in the evolution of prosociality and that complex cognitive capacities may not be a prerequisite for their expression.

A number of hypotheses have been put forward amongst which a species’ level of social tolerance (Massen et al. 2010; Tan and Hare 2013) and its dependence on cooperative behaviours such as cooperative breeding (Silk et al. 2005; Burkart et al. 2007) have been suggested to drive prosocial behaviour. Additionally a recent study suggests that in primates, differences in the propensity towards prosociality may be linked to the presence and extent of allomaternal care (Burkart et al. 2014). Such hypotheses would, however, benefit from confirmation in other taxa, since both allomaternal care and cooperative breeding are relatively widespread traits in other taxa and are actually rather limited in primates. Indeed, testing hypotheses about the potential variables affecting prosociality only in primates has its limitations. For example, Tomasello et al. (2012) proposed that one of the key elements of the evolution of human altruism was a dependence on collaborative foraging. However, collaborative foraging (e.g. cooperative hunting) occurs only sporadically in primates, making it difficult to include this variable in any predictive model when only primates are taken into account.

Considering the above, broadening the spectrum of species studied, adopting a comparative approach across closely related species with differing socio-ecological niches, and widely divergent species with convergent social structures or ecological niches, may be the best approach to further our understanding of what the ‘preconditions’ for the evolution of prosociality may be and what factors may affect its prevalence. Similarly, the inclusion of a more varied sample of species may allow us to probe questions regarding the cognitive requirements for such behaviours to manifest, and the underlying emotional mechanisms driving them (see below the insightful discussions sparked by rat and ant studies). However, ‘fair’ comparative studies across different species depend on the possibility of presenting comparable tasks to animals with different morphological features and cognitive abilities. Hence the challenge is to devise valid tasks, assessing prosocial tendencies, which can be easily presented to such different species as elephants, jackdaws and dogs and which are within their cognitive capacities. Here we try to make this task easier by reviewing the present literature with a particular emphasis on non-primate species and on critically evaluating the various test paradigms and the employed controls.

In that light, the main focus of the current review is the experimental paradigms that have been devised and adopted to tackle the potential presence and prevalence, as well as the underlying mechanisms, of prosocial tendencies in non-human animal species, i.e. (1) the so-called prosocial choice test (PCT) (comprising the token exchange and bar-pulling tests) in which animals have a choice to benefit either just themselves or both themselves and a partner; (2) the ‘helping’ paradigm, in which an individual is given the possibility of performing an act that enables another to obtain their—otherwise unachievable—goal; and (3) the most naturalistic of such paradigms: the ‘food-sharing’ paradigm, in which an individual is given the possibility of sharing food with his/her conspecifics. The latter two (the helping paradigm and food sharing) may in principle also be considered altruistic since they entail an immediate cost to the actor. However, we are more interested here in an evaluation of the experimental paradigms per se, rather than an assessment of the costs the actions may or may not entail.

Differently from previous reviews (Jaeggi et al. 2010a; Cronin 2012; Silk and House 2011; Yamamoto and Takimoto 2012; Warneken and Tomasello 2009; Brown et al. 2004; Stevens and Gilby 2004), in the following pages we give an overview of all three experimental paradigms used to assess prosociality in non-human species, highlighting the strengths and weaknesses of each method and suggesting potential ways in which the latter may be overcome. As will become evident, most studies have focused on non-human primates; however, we argue that broadening the spectrum of species studied is essential to enhance our understanding of (1) the social and ecological conditions which may have led to a selection for prosocial tendencies (see also McAuliffe and Thornton 2015 for a critical review of this issue) and (2) the underlying mechanisms that drive an animals’ choice to act prosocially or not. Hence, wherever present, studies on non-primate species are highlighted. Furthermore, in line with the aim of encouraging a broader comparative approach to the topic of prosociality, particular emphasis is placed on the methodological issues that need to be taken into account to allow for a more comprehensive approach to this topic.

## Experimental paradigms

### Prosocial choice test

**Fig. 1:**
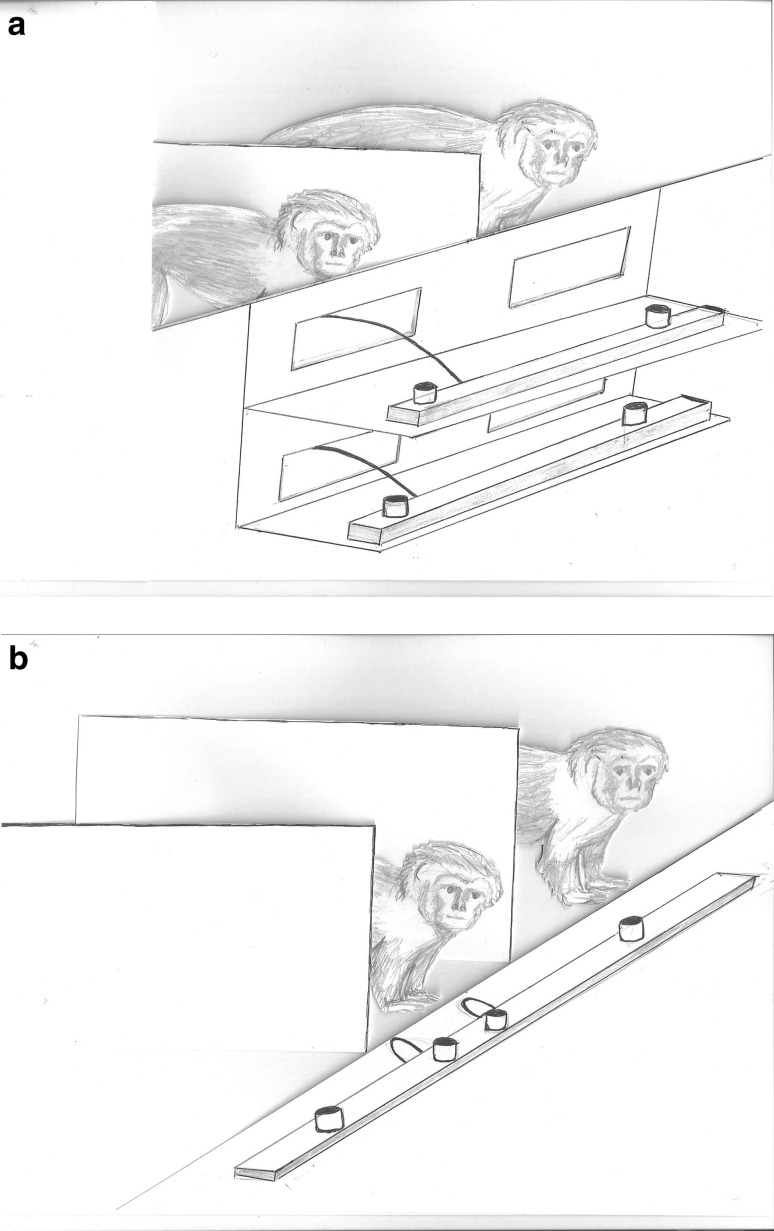
Two versions of the bar-pulling prosocial task with the food delivery trays placed either on top or adjacent to one another

A common means of investigating prosocial tendencies in non-humans is the prosocial choice test (PCT). In this test, subjects are typically given a choice between two reward combinations, one of which delivers a food item to the subject and their partner (prosocial choice) and the other which rewards only the subject (selfish choice; Colman et al. 1969). Thus the subject can opt to consider the partner’s welfare as well as their own or only to reward themselves (Fig. 1a). Typically, test sessions are compared with control conditions, which assess the actor’s choice (prosocial vs. selfish) when no partner is present.

Two main methodologies have been used to implement this test: token exchange and bar pulling. However, recently the PCT paradigm has also been adapted for use on a touch screen (Drayton and Santos 2014a).

The token exchange paradigm has been used to test a number of economic decision-making processes in non-humans (Brosnan et al. 2008), including inequity aversion (Brosnan et al. 2010a, b) and recently also prosocial behaviours. It requires a subject to return a non-food object to an experimenter in exchange for a food reward. In the test conditions, when a receiver is located in an adjacent compartment, actors can choose between two token types: prosocial or selfish. So far it has predominantly been used as a methodology with non-human primates, including great apes (Horner et al. 2011; Dufour et al. 2009; Pelè et al. 2009; Yamamoto and Tanaka 2010) and capuchins (de Waal et al. 2008; Skerry et al. 2011; Suchak and de Waal 2012) with one exception where it was used with parrots (Péron et al. 2012) (see Table 1).

**Table 1:** Prosocial choice task studies, including bar-pulling and token choice studies

Species	PC Test	Reward distribution (subject/partner)	Social effects	Effect of partner communication/visibility	Reward visibility/quality	Reciprocity	References
Chimpanzees	Bar-pull	1/0 versus 1/1	NA	None found	NA	None found	Silk et al. (2005)
Chimpanzees	Bar-pull	1/0 versus 1/1 0/0 versus 0/1	NA	None found	NA	NA	Jensen et al. (2006)
Chimpanzees	Kind of bar-pull	1/0 versus 1/1	NA	Partner reaching for a reward had no effect	NA	NA	Vonk et al. (2008)
Chimpanzees	Bar-pull	1/0 versus 1/1	NA	NA	NA	None found	Brosnan et al. (2009)
Chimpanzees	Bar-pull	Exp1: 0/0; 0/1; 1/0; 1/1; 1/3; 0/0 (1 on ground) = pull or not Exp2: 1/1 versus 1/0 1/1 versus 1/0 (1) 0/1 versus 1/0 1/1 versus 0/1	NA	NA	NA	NA	House et al. (2014)
Chimpanzees	Button choice	1/1 versus 1/0	No difference between mother–offspring and unrelated pairs	NA	NA	None found	Yamamoto and Tanaka (2010)
Chimpanzees	Token transfer	Subjects could transfer tokens to partner who could use them to obtain food Subjects could transfer food to partner	NA	Some effects of begging and social relationship-qualitative description	NA	Some effects found-exact results not reported	Nissen and Crawford (1936)
Chimpanzees	Token	1/0 versus 1/1	No effect of kin or dominance	More prosocial to neutral partners and partners reaching for food, but least prosocial to partners exhibiting begging/requests	Food not visible (but did not test if affected results)	None found	Horner et al. (2011)
Orangutans	Token transfer	Partner-valued tokens	NA	Reaching gestures resulted in more transfers	NA	Reciprocity found	Dufour et al. (2009)
Capuchin monkeys	Bar-pull	1/0 versus 1/1	NA	None found	If the recipient could get a better food than the donor, then donors more often chose the prosocial option	NA	Lakshminarayanan and Santos 2008
Capuchin monkeys	Bar-pull (one platform only)	Equal low (1/1 vs. 0/0) Equal high (1/1 vs. 0/0) Unequal (1/2 vs. 0/0) None (0/1 vs. 0/0 and 0/2 vs. 0/0)	NA	NA	Visibility made no difference	NA	Brosnan et al. (2010b)
Capuchin monkeys	Bar-pull	Middle/high versus middle/low value Or High/high versus high/low	Chose high value more for subordinate than dominant partners	Gave low value more when visual access to partner prevented	Donor received same reward, partner received high or low value	NA	Takimoto et al. (2010)
Capuchin monkeys (female)	Token	1/0 versus 1/1	More prosocial towards group members than strangers	Visibility of partner increased prosociality	NA	NA	de Waal et al. (2008)
Capuchins monkeys	Token transfer	Subjects could transfer tokens to partner who could use them to obtain food	NA	NA	NA	NA	Skerry et al. 2011
Capuchins monkeys	Token	1/0 versus 1/1	Prosocial rates in group same as out-group	NA	NA	None found	Suchak and de Waal (2012)
Capuchin monkeys	Touch screen symbols	1/1 versus 1/0 0/1 versus 0/0 1/0 versus 0/0 0/1 versus 1/1	NA	NA	NA	NA	Drayton and Santos (2014a)
Long-tailed macaques	Bar-pull	1/0 versus 1/1	More prosocial to kin than non-kin. Dominant more prosocial than subordinate individuals	NA	NA	NA	Massen et al. (2010)
Long-tailed macaques	Bar-pull	1/0 versus 1/1	No effect of relationship quality. Subordinates avoid giving to partners closest in rank	NA	NA	NA	Massen et al. (2011)
Stump-tailed and Rhesus macaques	Token	1/0 versus 1/1	NA	NA	NA	None found	Colman et al. (1969)
Rhesus macaques	Eye-tracking	1/0 versus 1/1 Or 0/1 versus 0/0	NA	NA	NA	NA	Chang et al. (2011)
Cotton-top tamarins	Bar-pull	1/0, 1/1, 0/1 or 0/0	NA	NA	Exp 3: subjects continued to pull when distribution was inequitable but subject got a reward. But stopped pulling when it was inequitable and subject received nothing	Gave more to trained monkeys who always gave, than to those who never gave	Hauser et al. (2003)
Cotton-top tamarins	Bar-pull	1/0 versus 1/1 0/0 versus 0/1	More prosocial towards mates than others	Partner reaching for a reward had no effect	NA	Na	Cronin et al. (2009)
Cotton-top tamarins	Bar-pull	1/0 versus 1/3 0/0 versus 0/3	More prosocial to cage-mates than strangers	NA	NA	NA	Stevens (2010)
Marmosets	Bar-pull	0/0 versus 0/1	More prosocial towards kin than non-kin. Male and female breeder as well as male helpers more prosocial than female helpers	Partner reaching for a reward had no effect	NA	NA	Burkart et al. (2007)
Macaques Capuchins Marmosets	Bar-pull in group setting	0/1-choice of pulling or not	Macaques: no effect of dominance Capuchins: NA Marmosets: no dyadic specificity for recipient	Macaques: NA Capuchins: NA Marmosets: no begging. Recipient reaching for the food had a negative effect on subject pulling	NA	NA	Burkart and van Schaik (2013)
Chimpanzees Bonobos Orangutans Spider monkeys Capuchin monkeys	Bar-pull and Token choice	Bar-pull: 1/0 versus 1/3 Token choice: 1/0 versus 1/1	NA	NA	Bar-pull: chimpanzees and spider monkeys selected prosocial more in control than experimental sessions when rewards were unequal	NA	Amici et al. (2014)
15 non-human primate species	Bar-pull	0/1 (pull or not)	Some effects of social bonds but extent of allomaternal care best predictor of prosociality	NA	NA	NA	Burkart et al. (2014)
Chimpanzees bonobos orangutans gorillas	Token transfer	Self-valued, partner-valued or no-value tokens available to both subjects-token transfers possible	No effect of affiliation	In orangutans, when the partner pointed, 80 % were followed by a transfer of a valuable token from the actor	NA	None found	Pelè et al. (2009)
Grey parrots	Token exchange	1/1 versus 1/0 versus 0/1 versus 0/0	The dominant bird shared but only reciprocally	NA	Food not visible (but did not test if affected results)	Some effects of reciprocity with conspecific and human partners	Péron et al. (2013)
Jackdaws	Bar-pull	1/0 versus 1/1 Or 0/1 versus 1/0	None found	More prosocial choices when recipients approached the food	NA	NA	Schwab et al. (2012)
Dogs	Bar-pull	0/1 versus 0/0	More prosocial towards familiar partners than strangers	None found	NA	NA	Quervel-Chaumette et al. (2015)
Ravens	Bar-pull	1/10 versus 1/1	NA	NA	NA	None found	Di Lascio et al. (2013)

*NA* where a particular issues was not tested for

The bar-pull apparatus usually consists of two movable shelves, either one on top of the other or side by side (Fig. 1a, b), placed in front of the subjects. The shelves are baited with food and can be moved into reach of both subjects when the bar is pulled. The bar can only be pulled by the actor and typically one shelf is baited with food exclusively for the actor (selfish) and the other shelf is baited with food for both animals (prosocial). This paradigm has seen more extensive use with primate species than the token exchange method, and versions of it have been extended to at least one non-primate species. Species studied include chimpanzees (Silk et al. 2005; Jensen et al. 2006; Brosnan et al. 2009; Vonk et al. 2008), macaques (Massen et al. 2010, 2011), capuchins (Lakshminarayanan and Santos 2008; Brosnan et al. 2010b), tamarins (Stevens 2010; Cronin et al. 2009), marmosets (Burkart et al. 2007) and jackdaws (Schwab et al. 2012); for more details see Table 1. More recently an extinction-type version of the bar-pulling task, where animals can choose when to stop pulling for their mates, has also been presented to dogs (Quervel-Chaumette et al. 2015) and rats (Rutte and Taborsky 2007, 2008; Schneeberger et al. 2012) and to a variety of primate species in a group setting, allowing wider comparisons to be made (Burkart and van Schaik 2013; Burkart et al. 2014).

However, both the token and the bar-pull versions of the prosocial choice test are open to a number of potential criticisms, which need to be taken into account when designing the task and appropriate control conditions, in particular when looking to adopt these to test more diverse species. In the following paragraphs, we outline some of the main concerns, how these have been addressed, and we make suggestions what may be done to further improve the PCT and its use with species beyond primates.

#### Understanding task contingencies

For both PCT methods (token choice and bar-pulling), a number of issues pertaining to the task set-up emerge: (1) over-training versus ensuring task comprehension; (2) paying attention to the partner; (3) the potentially distracting effect of food visibility.

1. Overtraining versus task comprehension.

The first issue is the difficulty in finding a good balance between allowing animals enough experience to *understand the mechanics of the task,* yet avoiding *over*-*training* the animals which may result in an inflated estimation of the prosocial choice during testing (see below). After basic training of pulling one bar or exchanging one token for food, typically training has involved just a few trials where the subject simply experiences the outcome of their choices (Table 2). However, in order to obtain clear results about prosocial preferences, it is important to ensure that the subjects understand the contingencies of the task. Indeed for both tasks, certain cognitive prerequisites are necessary for subjects to understand what is going on. In the more ‘intuitive’ of the two, i.e. the bar-pulling task, subjects must have at least some means-end understanding to know which bar brings which food reward(s) within reach of themselves and their partner. In the token tasks, the subjects must fully understand the meaning of each token, which requires learning and memory in order to associate the abstract token with a reward combination. It is therefore surprising that so many studies (see Table 2 for details) simply give the subjects experience of the outcomes of the different choices, without actually testing their understanding.

**Table 2:** A brief description of training and/or knowledge testing for studies using the PCT

Species	PC Test	References	Training	Knowledge test?
Chimpanzees	Token exchange	Nissen and Crawford (1936)	Not stated	No
Chimpanzees	Bar-pull	Silk et al. (2005)	Trained to pull the option that contained food All subjects participated in 2 trials as the recipient prior to testing	No but checked for a bias for the tray with more food items
Chimpanzees	Bar-pull	Jensen et al. (2006)	Training manipulation of the apparatus with two unconnected ropes allowing both tables to be pulled, then the test used a single rope, such that only one table could be pulled within reach	Yes: all four cups were baited and the door between actor and recipient rooms was open. Six trials were presented randomly between control sessions
Chimpanzees	Kind of bar-pull	Vonk et al. (2008)	Subjects were first trained to dislodge and receive both rewards for themselves Subjects also observed demonstrations of a partner receiving a reward from the apparatus for 1 trial as subject and 1 trial as receiver	No
Chimpanzees	Bar-pull	Brosnan et al. (2009)	Same set-up as test; 1/1 versus 1/0, partner present. Subjects had to reach a criterion of pulling any bar on 8/10 trials	Yes: after testing-16 trials with food only on receiver side (0/1 vs. 0/0). Obtained the reward on 58 % of trials
Chimpanzees	Bar-pull	House et al. (2014)	Study 1: no Study 2: exposure: 40 counterbalanced trials of the 1/1 versus 0/1 condition	Yes: as test but with access to recipient reward No
Chimpanzees	Button choice	Yamamoto and Tanaka (2010)	Subjects had access to both subject and receiver enclosures. Criterion: choosing 1/1 significantly more than 1/0 in 3 sessions of 10 trials	Pre-test knowledge test: partner present. Knowledge demonstrated when they continued to choose the 1/1 option Post-test knowledge test: reward distribution for the buttons was reversed-measured whether subjects learnt the reversal
Chimpanzees	Token Exchange	Horner et al. (2011)	Exposure: 5 trials/token = 10 trials	No
Orangutans	Token transfer	Dufour et al. (2009)	Same subjects as Pelè et al. (2009), thus subjects received only 12 trials as a refresher	No
Capuchin monkeys	Bar-pull	Lakshminarayanan and Santos (2008)	Each shelf baited with high quality on one side and low quality on the other. Barrier between enclosures so should select high quality on proposer’s side (criteria: 80 % correct). Step 2: barrier open, should now select so as to maximize high quality reward (criteria: 80 % correct)	No
Capuchin monkeys	Bar-pull (one platform only)	Brosnan et al. (2010b)	5 min where both monkeys had access to both enclosures. Then 10 trials with subjects separated	No
Capuchin monkeys	Bar-pull	Takimoto et al. (2010)	Only one shelf, both subject and receiver side baited but only had access to subject side. Criterion: stop showing interest in receiver side on 5 trials Second stage-as above but partner present. Criterion: no aggression shown to partner on 5 trials	No
Capuchin monkeys (female)	Token Exchange	de Waal et al. (2008)	Exposure: 5 trials/token = 10 trials	No
Capuchins monkeys	Token transfer	Skerry et al. (2011)	No meanings to learn. Trained to pass a token to adjacent enclosure	No
Capuchins monkeys	Token Exchange	Suchak and de Waal (2012)	Exposure: 15 trials/token = 30 trials	Yes: after testing, checked whether, when the partition is open, subjects would choose the prosocial option and move directly to both enclosures to gain the reward
Capuchin monkeys	Touch screen symbols	Drayton and Santos (2014a)	16 sessions of 32 trials Selfish training: both reward tubes deliver into the subject’s enclosure and subjects can receive both rewards Empty training: one reward tube now placed in the empty receiver compartment so subjects only receive one reward	During testing included a ‘selfish control’—same as selfish training
Long-tailed macaques	Bar-pull	Massen et al. (2010)	Eight trials: both shelves baited but after one is selected the other is blocked	No
Long-tailed macaques	Bar-pull	Massen et al. (2011)	Subjects already familiar with apparatus	No
Stump-tailed and Rhesus macaques	Bar-pull	Colman et al. (1969)	1/3 of trials forced choice to give subjects experience of the consequences of both levers	No but pilot testing involved switching the reward distribution for the levers, subjects learned this reversal
Rhesus macaques	Eye-tracking	Chang et al. (2011)	Conditioning to fix gaze on stimuli on the screen	No
Cotton-top tamarins	Bar-pull	Hauser et al. (2003)	Barrier present/absent and food accessible/inaccessible. Criteria: 100 % pull shelf when food accessible	No
Cotton-top tamarins	Bar-pull	Cronin et al. (2009)	Food on one of four locations (upper/lower on actor/receiver side). Criteria: select baited shelf 17/20 trials	No
Cotton-top tamarins	Bar-pull	Stevens (2010)	Not stated	Yes: partner absent, no barrier condition where either both the actor and receiver sides were baited (correct at 99 %) or only the receiver side was baited (correct at 96 %)
Marmosets	Bar-pull	Burkart et al. (2007)	Food only on receiver side on one of the two shelves. Criteria: select rewarded shelf 10/12 trials	No
Macaques Capuchins Marmosets	Bar-pull	Burkart and van Schaik (2013)	Subjects learned to pull and hold the handle with one hand while taking the food with the other. In the test the food was no longer reachable by the subject	Yes: access to food bowl blocked
ChimpanzeesBonobos Orangutan Spider monkeys Capuchin monkeys	Bar-pull Token exchange	Amici et al. (2014)	Food only placed in one enclosure and donor had access to one or both enclosures Criterion: get the food for themselves on every trial (six per session) on two consecutive sessions Receiver present in the receiver enclosure and donor had access to one token at a time to exchange. They immediately experienced the meaning of each token (one session of 6 trials). No criterion	No Yes. Partner absent, no partition between the enclosures
15 non-human primate species	Bar-pull	Burkart et al. (2014)	Subjects learned to pull and hold the handle with one hand while taking the food with the other. In the test the food was no longer reachable by the subject	Yes: Access to food bowl blocked
Chimpanzees bonobos orangutans gorillas	Token transfer	Pelè et al. (2009)	Criteria of exchanging 90 % self-value tokens first	No
Grey parrots	Token exchange	Péron et al. (2013)	3 trials/token = 12 trials demonstrated by a human	No
Jackdaws	Bar-pull	Schwab et al. (2012)	Step 1: one box baited (1/1) and the other empty (0/0). Criterion: choose baited box on 9/12 in 2 sessions Step 2: 0/1 versus 1/0, no access to recipient’s side Criterion: choose 1/0 on 9/12 in 2 sessions	No
Dogs	Bar-pull	Quervel-Chaumette et al. (2015)	Subjects were trained to pull the baited tray over the non-baited tray and gained the reward in the receiver enclosure. Criterion: choose baited tray on 17/20 trials in two sessions	Yes: after each test/control session the tray was baited in front of the subject’s enclosure and they were given the chance to pull to gain the reward for themselves
Ravens	Bar-pull	Di Lascio et al. (2013)	Phase 1: one box baited on the subject’s side (1/0) and the other one empty (0/0) Phase 2: one box baited on the subject’s side only (1/0) and the other box baited only on the recipient’s side (0/1) Phase 3: one box baited with one food item on the subject’s side (1/0) and the other box baited with 3 food items on the recipient’s side (0/3)	Yes: in the training phases, authors checked whether ravens understood the contingencies of the task. They also checked whether the choices were based on the number of food item visible (see phase 3 of the training). Included also “attention trials” during test phase where the subjects could choose between a box only baited on the subject’s side (1/0) or a box only baited on the recipient’s side (0/1)

This criticism is particularly pertinent to the token exchange studies where it is often assumed that the meanings of the tokens are understood after as few as 10 trials (e.g. de Waal et al. 2008; Amici et al. 2014). This minimal training in fact resulted in de Waal et al. (2008) analysing only the final 10 trials of the test as the subjects changed their behaviour during the first two sessions, presumably as a result of their increased understanding of the contingencies of the task.

In order to meet that criticism, a number of studies have incorporated controls to ensure task comprehension. For example, Burkart et al. (2007), Cronin et al. (2009) and Lakshminarayanan and Santos (2008) all used a criterion in their training sessions to ensure the animals were paying attention to which shelf was being baited (see Table 2 for an overview of training methods and criteria). However, in many of these studies in which conditions ensured subjects understood the task prior to testing, a side effect may have been an over-training of the ‘prosocial’ option, since during training animals were rewarded for choosing the option that then delivered food to their partner during testing. This over-training could thus have resulted in an overestimation of prosocial behaviours. Researchers have countered such a possibility by including control conditions, typically allowing subjects to pull for an ‘empty’ enclosure versus the enclosure with the partner within it (e.g. Lakshminarayanan and Santos 2008; Cronin et al. 2009). However, in a number of studies no differences emerged between such control and test conditions (e.g. Cronin et al. 2009), or effects were rather weak (e.g. Lakshminarayanan and Santos 2008: significantly different only when applying a one-tailed level of significance). But over-training may in fact also mask such differences, since subjects may continue to perform the trained action in all conditions, lacking the inhibitory capacity to refrain from carrying out a previously reinforced behaviour.

An alternative approach to avoid ‘over-training’, has been to carry out ‘knowledge’ tests, in which the subject’s understanding of the task contingencies are assessed either during or at the end of the prosocial testing phase (Brosnan et al. 2009; Suchak and de Waal 2012; Stevens 2010; Jensen et al. 2006; Drayton and Santos 2014a) thereby avoiding the potential carry over effects from training into testing. Typically in such tests the reward distribution and or access to the enclosure in which the food is delivered are changed, so that subjects have the choice to access the food themselves (see Table 2). These knowledge tests have proven important since in at least one of the studies (Brosnan et al. 2009), chimpanzees that had access to the food in the adjacent enclosure and thus could deliver food for themselves only chose the correct tray delivering the food on 58 % of trials, indicating a somewhat incomplete understanding of the task contingencies.

2. Attention to the partner.

The second methodological issue that needs to be addressed in such tasks is how to guarantee that subjects pay attention to the consequences their actions have for the partner. To address this issue a number of authors (Lakshminarayanan and Santos 2008; Horner et al. 2011) *manipulated the reward distribution* in terms of its quantity and/or quality. In a bar-pulling task, Lakshminarayanan and Santos (2008) showed that subjects chose the prosocial option even more when their own outcome was of lower quality than their partners. The authors interpreted this data as resulting from a better attentiveness to the location of the high-quality food when the subjects themselves received only low quality food. However, House et al. (2014; study 2) found that chimpanzee donors were equally indifferent to payoffs obtained by their partner regardless of their own reward outcome (0/1 vs. 0/0 and 1/1 vs. 1/0). Surprisingly few studies have included behavioural coding of the subject’s looking behaviour to the partner (de Waal et al. 2008; Quervel-Chaumette et al. 2015), which would be one way to evaluate their level of attention to the partner’s outcome. This aspect could be easily included in future studies.

3. Food visibility.

The final methodological issue for the PCT regards food visibility. The bar-pulling task typically allows subjects to see the food distribution, whereas in the token task the food is not in sight. Warneken et al. (2007) suggest that the use of food rewards may obscure the propensity for prosocial behaviour because subjects may treat all interactions involving food as a competitive situation (see also Cronin 2012). Indeed in the token exchange task, Horner et al. (2011) found that chimpanzees were more willing to show prosociality if the reward was invisible (i.e. wrapped in paper), potentially because animals did not need to control or refrain their immediate impulse to eat the food and hence could be more attentive to the task and their partner. However, Brosnan et al. (2010b) replaced the food rewards on the bar-pulling shelves with tokens but did not find a prosocial effect with capuchins using this modified version. Furthermore, the higher prosocial choice shown by chimpanzees when the food was wrapped in the Horner et al. (2011) study may have been a result of associative learning of reward contingencies whereby the noise of the wrapper acted as a secondary reinforcer associated with food delivery (Heyes 2012). Remarkably only one study has used both the bar-pulling task and the token choice with the same animals (Amici et al. 2014). Prosociality was not elicited in either paradigm in this study, making it difficult to draw conclusions on the effect of the two methodologies on the prosocial tendencies. Brosnan et al. (2010b) also investigated the effect of food visibility by including a ‘token treatment’ in their bar-pulling study, whereby subjects could pull a shelf containing a token, which could then be exchanged for food. Unlike the food-visible treatment, Capuchins did not differentiate between the control and partner-present conditions in the token treatment, although it is not clear from the results of this study why this may have been the case.

In an attempt to address both the issue of task comprehension and attention to the partner’s outcome, Burkart et al. (2007) adopted a simpler paradigm first with marmosets and more recently in a group setting with a variety of primate species (‘group service’ paradigm; Burkart and van Schaik 2013; Burkart et al. 2014). In the dyadic version of this set-up, the subject can choose whether to pull a tray delivering no food at all or food only to the partner (0/0 vs. 0/1). The task is simpler because the subject needs to keep track of only one food item and simply choose whether or not to deliver it depending on who is in the adjacent enclosure (partner vs. none and/or varying the identity of the partner). However, as highlighted by Thornton and McAuliffe (2015), Burkart et al. (2014) did not randomize the sequence of the conditions but always presented the control condition (i.e. 0/1 but with a mesh blocking the food delivery to the partners) after the experimental condition. Although subjects pulled more in the experimental than the control condition, leading the authors to conclude that they exhibited behaviour driven by a prosocial concern, a simpler explanation such as a decrease in motivation in later trials cannot be excluded.

Overall, these examples highlight the need to ensure subjects are paying attention to the outcome of their actions and incorporate conditions in which the animals’ understanding of the task is assessed, while at the same time avoiding the potential pitfalls of over-training subjects’ behavioural response. This would appear to be even more crucial in light of the need to extend studies beyond primates to less studied species, for which we may have a more limited understanding of their cognitive abilities, and/or the speed with which they may associate their actions to specific outcomes.

#### The effect of social relationships and partner interaction

When evaluating prosocial tendencies, the *behaviour between individuals during the test* and the *social relationshipbetween partners* have also been shown to be crucial.

In regard to the first, a number of authors report that the *visibility of the partner* (de Waal et al. 2008; Horner et al. 2011) as well as *the communication between animals* during the test (in particular the recipient’s behaviour towards the actor) and the *possibility of recipients expressing behaviours that clarify their goal* (e.g. reaching for the food) are important factors which could influence the subject’s response (Silk et al. 2005; Burkart et al. 2007; Cronin et al. 2009). Communicative behaviours in particular have been suggested to show an understanding of the actor’s role in delivering the reward (Silk et al. 2005). However, although the occurrence of behaviours that make the partner’s desire for a specific outcome more explicit has been reported to increase the number of prosocial responses in some studies (Schwab et al. 2012; Pelé et al. 2009 and ‘helping’ studies—see below), this has not been the case for all (Burkart et al. 2007; Silk et al. 2005; Vonk et al. 2008; Cronin et al. 2009). Indeed Cronin et al. (2009) found the opposite trend, with marmoset actors providing fewer rewards to partners on trials during which their partner reached out for the reward compared to trials when they did not. Yet, although at present the evidence on the importance of communication between partners in the emergence of prosocial behaviour is inconclusive, it appears to be an important variable to keep in mind when designing experimental paradigms investigating this issue.

In the human studies of prosociality (mostly in the field of economic behaviour) that inspired the field of animal prosociality, the emphasis is on interactions among strangers (Engel 2011), but very few studies in the animal literature have included strangers in their paradigms (Quervel-Chaumette et al. 2015; Hernandez-Lallement et al. 2015), probably due to the potential risks involved in testing pairs of animals unknown to each other. However, some studies have addressed the potential effects of the closeness of the social relationship between tested partners, with current evidence suggesting that a strong social bond can make prosocial choices more likely (Cronin 2012). Examples include Chang et al. (2011), who used eye-tracking, and de Waal et al. (2008), using token exchange. However, there are contrary examples, for example Yamamoto and Tanaka (2010, token exchange) and Stevens (2010, bar pulling). Furthermore, many species tested form stable social dominance hierarchies, and rank has also been shown to have an effect on prosocial choices. In a number of studies prosociality has been shown to be more likely to occur down the hierarchy, i.e. from dominant to subordinate individuals (Cronin 2012). Examples include studies using token exchange in chimpanzees (Horner et al. 2011), and bar-pulling in capuchins (Takimoto et al. 2010; Lakshminarayanan and Santos 2008) and macaques (Massen et al. 2010). However, this trend has not been replicated with other paradigms, and in some cases the opposite has been found (e.g. in helping studies: Yamamoto et al. 2009; Melis et al. 2011).

**Fig. 2:**
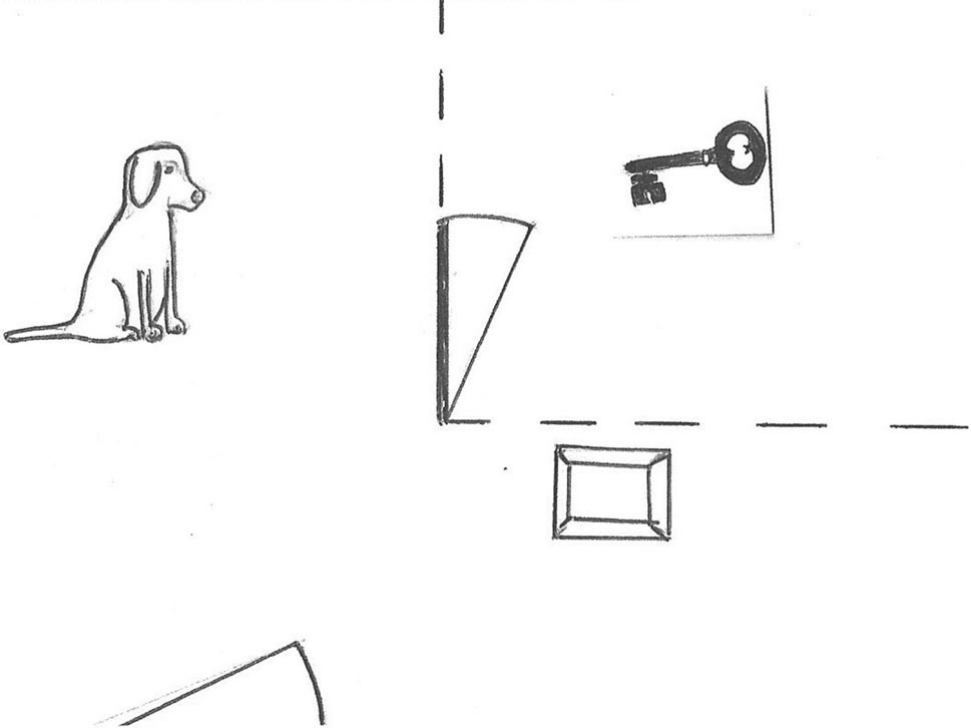
Schematic depiction of the Bräuer et al. (2013) study. Dogs could press a button on the ground to open the door, to allow the person to retrieve their key

Regardless of results, studies looking at the likelihood of prosociality occurring towards specific individuals have needed, more than others, to contend with a number of potentially confounding variables (e.g. socially influenced side biases). In their experiments, Massen et al. (2010; bar-pulling) and de Waal et al. (2008; token exchange) concluded that primates were more prosocial towards kin or friends than non-kin or non-group members. However, the apparatus used for these studies raises questions about potential side biases, a problem also highlighted by Jensen et al. (2006). De Waal et al. (2008) state that a number of subjects (but the exact number is not reported, p. 13,689) showed a preference for a token placed on one side rather than the other, potentially affecting the overall pattern of results. Massen et al.’s (2010) set-up could allow animals to show a preference for sitting close to a friend/kin rather than a non-friend/kin. Pulling the rope closest to them would then result in a seemingly prosocial choice directed at the specific target, but it could have been just a by-product of their initial location preference. Massen et al. (2010) followed up on this limitation and found that the amount of time subjects spent in each area of the testing room did not correlate with the bar choices in the test; however, alternative and more convincing set-ups to avoid this issue have been devised. One approach is to use top/bottom shelves or a triadic choice set-up with the bar-pulling task, as shown in Figs. 1 and 2. Subjects used with this procedure have included chimpanzees (Silk et al. 2005), marmosets (Burkart et al. 2007), tamarins (Cronin et al. 2009), capuchins (Lakshminarayanan and Santos 2008) and macaques (Massen et al. 2011). A second approach is to place all token types together in a box (as used with chimpanzees by Horner et al. 2011), to ensure that the location of choice remains constant despite the changing partners. Recently, Amici et al. (2014) also highlighted the limitations of Massen et al.’s (2010) study and included an ‘equidistant condition’ in their own study where the set-up was the same, but the bars were placed centrally in front of the subject so that prosocial or selfish choices were not dependent on whether the subject preferred to approach or avoid the partner’s compartment.

Given that overall results show the importance of the partner’s identity and actions on the subject’s prosocial behaviour, the issue that arises is what is the most appropriate control condition for the prosocial test? Indeed in all PCT studies, the main control condition involves the ‘receiver’ compartment being empty, with the partner being completely absent from the testing room/enclosure. However, from the social learning literature it emerges that the likelihood of a behaviour being performed (particularly involving food) may be increased by the mere presence of a social partner, often referred to as ‘social facilitation’ (Dindo et al. 2009; Glickman et al. 1997). Hence, a more stringent control condition would involve a social partner being present, but in a location where the food cannot be delivered. So far only two studies, to our knowledge, included such a control condition: in Jensen et al. (2006) chimpanzees were shown to be equally ‘prosocial’ when the partner could obtain the reward compared to when the partner was present but could not access it, whereas in Quervel-Chaumette et al. (2015) dogs delivered more food to familiar partners when they could obtain it than when they were present but in a location which did not allow them to reach the food. Implementation of social facilitation control condition would appear to be essential to guarantee validity of prosocial results.

#### PCT in non-primates

The classic PCT, i.e. testing the prosocial versus selfish (i.e. 1/1 vs. 1/0) choice of a subject, has been used with only a few non-primate species, namely jackdaws (Schwab et al. 2012, bar pulling), ravens (Di Lascio et al. 2013), parrots (Péron et al. 2012, token choice) and rats (Hernandez-Lallement et al. 2015, using a location choice variation of the task). In addition, a simplified version of the PCT (i.e. 0/0 vs. 0/1) was recently used by the current authors in a bar-pulling study with pet dogs (Quervel-Chaumette et al. 2015).

Schwab et al. (2012) used a bar pulling paradigm but with the subjects facing one another rather than being placed side-by-side (although see Takimoto et al. 2010). The authors found that jackdaws provided significantly more food to a conspecific of the opposite sex than one of the same sex, but only in trials where the partner approached the food resource before the subject made their choice. Considering that no begging or other forms of direct requests were exhibited by partners towards the subject, the authors concluded that it was probably local/stimulus enhancement mechanisms that directed the subject’s behaviour towards the apparatus, rather than prosocial tendencies per se.

A modified version of the bar-pulling task has been used with ravens (Di Lascio et al. 2013). The birds did not, however, show any prosocial behaviour towards conspecifics. Interestingly, the authors included both attention trials, to ensure that animals followed the food distribution in the task, and a control condition to test for the animals’ understanding of the set-up. Indeed, the latter condition proved to be crucial, since authors concluded that given the random performance of the animals in these trials, little could be concluded as regards their other regarding preference in test trials.

Péron et al. (2012) allowed two grey parrots to choose between prosocial, selfish, non-rewarded or giving (only the partner is rewarded) tokens. The parrots stopped selecting the non-rewarded token in all conditions, which suggests that the animals had some understanding of the task and the token meanings. Furthermore, when paired with a selfish or generous human partner, subjects adapted their choices accordingly by increasing selfish choices for the selfish partner and increasing prosocial choices for the giving partner. This study, however, does have serious limitations including significant side biases in the subject’s choices, which were not controlled for in the analyses, and vastly different patterns of choices carried out by the two individuals participating in the study. But, although firm conclusions cannot be drawn, it does indicate that the PCT may potentially be used with parrots.

Rats have recently been tested in a modified version of the PCT where rather than pulling a tray or selecting a token, subjects could choose between entering one of two chambers leading to different outcomes (Hernandez-Lallement et al. 2015). Entering one chamber consistently resulted in both the subject and their partner being fed in adjacent chambers. Entering the alternative chamber, resulted in the subject being fed, but the partner, present in the adjacent chamber was not given any food. The control condition involved a dummy rat being placed in the chambers instead of the real-life partner. Rats were significantly more prosocial with a real-life partner than a dummy, and they chose the prosocial choice above chance (55 %). However, as the authors point out there was a large individual variability among subjects, and prosocial choices appeared to be influenced by the body weight perception of the partner, an indicator of status (dominance) in rats. Interestingly, in the current study actor and partner rats were strangers to each other until testing, and they never met (except in adjacent chambers during testing). Hence the current study suggests that it is not just the established relationship between individuals, which may affect prosocial tendencies (as has been shown with primates), but also the perception of the other’s status when the partner is a stranger.

Finally, Quervel-Chaumette et al. (2015) adopted a simplified version of the bar-pulling task (i.e. 0/0 vs. 0/1 see Burkart et al. 2007) to test the prosocial tendencies of pet dogs, and whether these would vary depending on the familiarity of the receiver. We adopted an extinction-type paradigm, in which dogs were first trained to pull the tray to obtain food for themselves, then we assessed whether they would stop their pulling behaviour at different rates depending on the condition presented. Considering the potential social facilitation effects on the likelihood of performing a learned action, control conditions included the classic ‘empty’ enclosure, but also conditions in which the partner was present, equidistant to the subject, but in a location which did not allow them to gain access to the reward. Results showed that dogs continued delivering food to the familiar partner for longer in the test compared to both the social facilitation and empty enclosure control conditions. Furthermore, subjects delivered food for longer to the familiar than the stranger partner, but the behaviour of the partner (in terms of attention-getting behaviours towards the subject or attempts to get the food from the apparatus) did not affect the giving rate of the subject. Subjects’ comprehension of the task was further assessed by including ‘knowledge’ trials at the end of each test and control session; in these trials the food location on the apparatus changed, so that subjects could now pull the tray to obtain food themselves. In all such trials, subjects resumed pulling.

The studies outlined above suggest that with some modification in order to simplify the task (e.g. using ‘choice of location’ as in Hernandez-Lallement et al. 2015 or an easier food distribution layout as in Quervel-Chaumette et al. 2015), the PCT may also be applied to non-primate species, opening up the possibility for a much needed broader range of comparative research on prosociality.

However, it is important to note that the version of the bar-pulling used in the study with dogs (Quervel-Chaumette et al. 2015) changed the distribution of reward from the classic selfish versus prosocial choice (1/0 vs. 1/1), to a distribution which no longer rewards the actor for its actions and only delivers food to the receiver (0/0 vs. 0/1). Hence, based on a number of definitions, this version of the PCT may be considered more a test of ‘altruism’ or ‘helping’, than prosociality, since it appears to extract an immediate cost from the actor. From a motivational point of view these differences may be critical, in that the choice to deliver food to another with no immediate reward for oneself may potentially indicate a more prosocial motivation. However, where direct comparison of different reward distributions have been carried out (e.g. Jensen et al. 2006; House et al. 2014), results suggest that the more complex the task (i.e. the more food items present that need tracking) the smaller the prosocial response (House et al. 2012, 2014).

### Helping experimental paradigms

Helping has been defined as a form of cooperation that involves immediate costs for the actor and yields benefits *exclusively* to the recipient (at the proximate level), hence it is not just prosocial but also altruistic (see introduction above). Most studies of helping behaviour have been carried out with chimpanzees and capuchin monkeys (see Table 3), using paradigms in which one individual is given the possibility of performing an act that enables another individual to obtain their—otherwise unachievable—goal (‘instrumental helping’ Warneken and Tomasello 2009 or ‘targeted helping’ de Waal 2008).

**Table 3:** ‘Helping’ studies

Species	Partner	Task	Social effects	Effect of partner communication/visibility	Food visibility	Reciprocity	References
Humans Chimpanzees	Human	Helping/Object transfer	NA	Both species helped but chimpanzees less so (perhaps due to less understanding of the partner’s goal)	NA	NA	Warneken and Tomasello (2006)
Humans Chimpanzees	Human	Helping/Object transfer	NA	Both species helped but chimpanzees needed more communicative cues by the partner	NA	NA	Warneken et al. (2007) Experiment 1 and 2
Chimpanzees	Conspecific	Helping/opening a door	NA	Helped more when partner close to target object/location	NA	NA	Warneken et al. (2007) Experiment 3
Chimpanzees	Conspecific	Helping/Object transfer	Subordinate helped more than dominant	Helped more when partner exhibited begging	NA	None found	Yamamoto et al. 2009
Chimpanzees	Conspecific	Helping/Bar-pull	NA	Helped conspecifics (after having obtained food themselves). No effect of partner communication	NA	NA	Greenberg et al. (2010)
Chimpanzees	Conspecific	Helping/releasing a food delivery mechanism	No effect of dominance	Helped more when partner exhibited noisy attention-getters	Token versus food: no effect	NA	Melis et al. (2011)
Capuchins	Human	Helping/Object transfer	NA	Helped only if rewarded, no effect of partner communication	NA	NA	Barnes et al. (2008)
Capuchins	Conspecific	Helping/Object transfer	NA	No effect of partner presence on token transfer	NA	NA	Skerry et al. (2011)
Capuchins	Human	Helping/Object transfer	NA	Two objects present. Communication strongly affected the choice of the transfered object	NA	NA	Drayton and Santos (2014b)
Dogs	Human	Helping/Showing	NA	Did not help/show where the object is if only the human showed interest in it	NA	NA	Kaminski et al. (2011)
Dogs	Human	Helping	NA	Helped/opened the door for the human only if human reached spontaneously for the target or pointed at the release button	NA	NA	Bräuer et al. (2013)
Rats	Conspecific	Helping	NA	Helped/released their partner from cage more than in control (no trapped partner). Also when incurring additional cost (sharing food)	NA	NA	Ben-Ami Bartal et al. (2011)
Rats	Conspecific	Helping	Rats response driven by desire for social contact not ‘empathy’	Helped/released partner from cage but mostly if it provided them with social contact	NA	NA	Silberberg et al. (2013)
Ravens	Conspecific	Helping/Object transfer	NA	No effect	NA	NA	Massen et al. (2015)

*NA* where a particular issues was not tested for

A number of studies with primates have adopted the ‘out-of-reach’ paradigm in which either a human or a conspecific can be helped to obtain an object/food it cannot reach by a subject that can fetch the object (Warneken and Tomasello 2006; Warneken et al. 2007; Barnes et al. 2008; Yamamoto et al. 2009; Drayton and Santos 2014b), open a door (Warneken et al. 2007) or activate a delivery system (Melis et al. 2011) (Table 3).

In most studies with a human as a recipient, both toddlers and chimpanzees retrieved the out-of-reach object more in the experimental condition when the subject observed the recipient exhibiting clear behavioural signs of wanting to reach the specific goal, than in the control condition, when no such behavioural signs were exhibited. Crucially, however, toddlers retrieved the object without the need for additional cuing by the recipient, but the chimpanzee population tested required recipients to call out their names and attract their attention in various ways (Warneken and Tomasello 2006; Warneken et al. 2006, 2007), and capuchin monkey only retrieved the object if a food reward was exchanged for it (Barnes et al. 2008).

In most studies with chimpanzees (except Greenberg et al. 2010, who used a modified version of the bar-pulling task), clear behavioural cues by the recipient appeared to play a crucial role in encouraging ‘helping behaviour’ towards conspecifics. Helping occurred more often when the receiver was attempting to obtain its goal or when exhibiting behaviours apparently aimed at getting the actor’s attention (Warneken et al. 2007; Melis et al. 2011; Yamamoto et al. 2009).

There are two possible explanations why chimpanzees may help more when their partner’s behaviours are more pronounced. One possibility is that these behaviours make the partner’s aim clearer to the subject, who is then more willing to help. The alternative explanation is that the partner’s behaviours act to enhance the stimulus (for example the object that has to be handed over, or the peg that needs to be removed to release the food), which may increase the likelihood that the subject will interact with it.

Both Warneken et al. (2007) and Melis et al. (2011) argued for the former explanation, maintaining that the subject’s actions in their helping studies were more likely elicited by the communicative acts of the receivers because these led to an understanding of their goal-directed actions. Melis et al. (2011) further added that ‘stimulus enhancement processes’ could be excluded since in object retrieval studies the subject does not just take up the object and manipulate it but actually carries and hands it over to the experimenter. However, when the recipient of the object is a human this may be heavily dependent on prior experience both within and outside ‘scientific testing’, which is rarely explicitly stated. Had animals participated in token/tool exchange tasks before? Was handing over objects to obtain a treat a common occurrence in their husbandry, and hence rewarded in some way?

Recognizing some of these issues as potentially problematic, Drayton and Santos (2014b) adopted a more promising approach with capuchin monkeys, in that two objects where always simultaneously present in the test enclosure, but only one was the target of the experimenter’s communicative actions. Capuchin monkeys reliably handed the ‘correct’ object over when the experimenter acted out that she desired it in various ways (i.e. moving and static reaching). However, the authors themselves caution that it remains to be tested whether the monkeys were indeed understanding something of the experimenter’s goal, or rather simply perceiving the experimenter’s actions as object-directed and hence enhancing the salience of the desired object. The latter may be even more probable if an animal has had prior experience in handing over objects to caretakers in their daily keeping routine, an issue that is rarely mentioned in such studies.

In Melis et al’s study (2011), chimpanzees learned to activate a food delivery system initially for themselves. In test trials they no longer had access to the receiver room, which was instead occupied by a partner. In some trials the partner could noisily rattle the chain that needed to be pulled by the partner, and in other trials it could not. In a further control condition the partner was in an enclosure that did not allow access to the food. Subjects delivered food to the partner more when he actively rattled the chain. But they also delivered food to the same extent when the partner was in the recipient enclosure (had access to the food, but did not rattle the chain) and when he was in the furthest enclosure (when they had no access to the food). It is not possible therefore to tease apart whether the subject’s action were elicited simply by the increased salience of the object or whether the presence of the partner in the correct enclosure was an indispensable factor. The crucial control would have been to increase salience of the object at a time when the partner could not receive the reward.

Along these lines, two studies, one with capuchin monkeys, the other with chimpanzees, suggest that enhancing the salience of the object rather than clarifying the goals of the recipient may be the more important aspect influencing subject’s behaviours in ‘helping paradigms’. In Skerry et al.’s (2011) study a capuchin monkey could donate tokens to its companion who had access to the ‘vending machine’ from which they could obtain the food. In a series of control conditions, the authors aimed to distinguish whether it was the perception of the end state (token in vending machine) that elicited the token transfer or the perception of the recipient’s goal (presence/absence of the partner). Results showed that it was the presence of the vending machine that affected transfer rates, while the presence of the partner in the adjacent enclosure did not, in that animals dropped tokens in the empty enclosure just as often with and without the partner in it. In Yamamoto et al.’s (2009) study, a chimpanzee could hand over a tool to its partner who had access to a juice box (but no tool). The authors recorded the partner’s behaviours directed at the tool or the juice box (i.e. their goal) and those directed at the subject and found that it was the actions directed at the subject, and not those directed at the goal, that elicited helping behaviour (i.e. transfer of the appropriate tool). Hence results from this study suggest that the communicative acts may have functioned more as attention-getters rather than as a clarification of the receivers’ goals.

Overall, whether the donor’s helping behaviour is elicited by an understanding of the recipient’s goal, or whether requesting gestures function as an attention-getter or simply increase the salience of the object, remains an open question. In studies involving object transfer between conspecifics (e.g. Yamamoto et al. 2009), i.e. where prior training to hand over an object may not be an issue, the act may be considered prosocial regardless of the subject’s behaviour to elicit it, since the subject should have no expectation that handing over the object to the partner will result in reward for itself. However, in studies where the behaviour is learned/trained (e.g. removing a peg to obtain food, Melis et al. 2011) or encouraged during daily routines (e.g. handing objects over to the caretaker), enhancing the salience of the object and/or the receiver may act as a ‘prime’ for the performance of the familiar (previously rewarded) action, which would hence have little to do with a prosocial motivation to act. In general the previous experience of animals not just in the testing context but also in their everyday routines needs to be taken into account in task design.

#### ‘Helping’ studies in non-primates

Helping behaviour in non-primate species has received very little attention. To our knowledge only four species have been tested experimentally: dogs (Kaminski et al. 2011; Bräuer et al. 2013), rats (Ben-Ami Bartal et al. 2011; Silberberg et al. 2013), ravens (Massen et al. 2015) and ants (Nowbahari et al 2009). Interestingly, in some of these studies, new experimental paradigms were used which sought to test for the underlying motivation guiding the animal’s ‘helpful’ action.

Bräuer et al. (2013) adapted the out-of-reach paradigm used in studies with chimpanzees by Melis and colleagues to assess dogs’ willingness to help a human partner. Dogs were initially trained to press a button, which opened a door to a fenced part of the room and obtain food from within. Subsequently, a set of keys were placed inside the fenced area and the experimenter expressed a desire to obtain them in various ways (e.g. by reaching towards them, or trying to open the door) (Fig. 2). As a control condition, food was placed in the target room and the experimenter paid no attention to it. Results showed that dogs opened the door significantly more in this control than in most other conditions. The only conditions in which they pressed the button to open the fence when keys were in the area was when the experimenter used pointing gestures towards the button, or used ‘natural reaching’ gestures towards the keys. On the basis of these results, the authors conclude that when the human was allowed to react more spontaneously to the dogs’ behaviours, the dogs understood the person’s goal better and therefore helped more than in the other conditions. However, the pointing gestures were directed at the button, so rather than clarifying the goal it may have given subjects an indication of what action they were required to perform. Furthermore, some comparisons were based on inadequate sample sizes; for example, the potential difference between experimenter and owner was based on three dogs (pp. 146). In the further analyses, despite this potential difference, the data from the owner and experimenter conditions were pooled when comparing dogs’ behaviours in the two crucial conditions, i.e. when the person showed no interest in the keys and when they did so in a ‘naturalistic’ manner. Accordingly, a more careful treatment of comparisons and larger sample sizes would be needed to confirm the results.

Kaminski et al. (2011) adopted a novel approach using dogs’ tendency to display ‘showing behaviours’ towards humans, i.e. dogs’ ability to indicate to humans the location of hidden food/objects (Miklósi et al. 2000). Interestingly in this paradigm, the authors attempted to distinguish whether dogs were able to take the ‘need’ or ‘desires’ of the individuals they were supposed to help into account. Hence the basic set-up involved an initial, preparatory phase in which either just a human or just the dog, or the two of them together, interacted with one of two objects. In this phase, the aim was to establish who had the ‘need’ or interest in the object. The objects were then hidden by another actor, while being watched by the dog but not by the human. When the human returned, they attempted to locate the object based on the dogs’ behaviour. Overall results suggest that dogs indicated what they themselves desired but not what their human partners wanted, most probably because they could not take the needs of their human partner into account.

Although the methodology used (i.e. showing behaviour) may be very specific to dogs and hence have limited applicability to other species, this study is particularly interesting in its attempt to assess the underlying prerequisites of helping behaviour rather than assuming them. Indeed, for a behaviour to be considered helpful, the subject has to show an awareness of their partner’s needs/desires/goals, and subsequently decide to meet them. Addressing these issues separately may be an important step forward for future research.

**Fig. 3:**
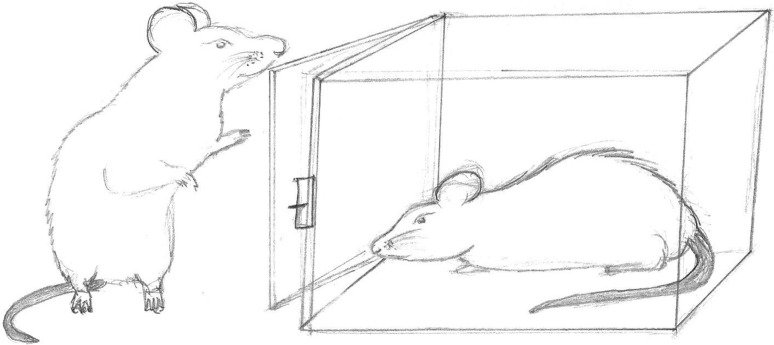
Paradigm used by Ben-Ami Bartal et al. (2011), a ‘trapped’ rat could be released by its partner

Ben-Ami Bartal et al. (2011) report findings of helping in rats. In their task, one rat was trapped in a small central tube while another was free to roam the larger enclosure around it (Fig. 3). Over time, free rats learned to open the door to release the trapped rat. Once they had learnt this, they did so consistently and with progressively shorter latencies. Furthermore, they opened the door significantly more than when the central tube was empty. Subsequently the rats were presented with two cages: one containing a trapped rat and the other containing chocolate chips. The rats in this condition opened both doors and, because they had to share the food reward, ate fewer chips than when there was only one cage containing chocolate chips. In sum, the authors concluded that rats were both empathic and prosocial towards their partner.

Although it is commendable that Ben-Ami Bartal et al. attempted to design a novel paradigm and included a number of controls, there is a major confound in their design. All the rats in the control conditions first experienced the test condition, and this may have impacted their door opening behaviour in later conditions. Silberberg et al. (2013) recently addressed this issue by testing naive rats on one key control condition. In their task, the trapped rat was released not into the same large enclosure, but into a separate area. This controls for the possibility that the rats are opening the door in order to obtain social contact, rather than for a prosocial concern for their partner. It was found that rats took significantly longer to open the door when this action did not result in social contact, than when the trapped rats were released into the same enclosure, supporting the social contact hypothesis. Additionally, door openings were correlated with the amount of time the free rat spent in the area of the tube suggesting that perhaps the door opening behaviour was not at all an intentional act but merely a by-product of proximity to the cage-mate (and thus, the tube). This argument is further supported by results from Silberberg et al.’s (2013) final control, where attempts to open the door did not diminish, despite the action never actually opening the door.

Nowbahari et al. (2009) also tested prosociality in terms of ‘rescuing behaviours’, but they focused on ants and tested whether such behaviours may be preferentially directed to kin. They found that ants (*Cataglyphis cursor*) will release active (non-anaesthetized) nest-mates restrained by a nylon snare in a sand field, but not (1) anaesthetized nest-mates, (2) ants from different colonies, (3) sympatric unrelated species or (4) prey items. Interestingly, the study highlights once again the potential occurrence of ‘helping’ behaviour which may not necessarily hinge on a psychological understanding of another’s goal or empathic like behaviour. Indeed in an insightful commentary Vasconcelos et al. (2012) contrast the Nowbahari study with ants and the Ben-Ami Bartal et al. study with rats, highlighting that despite the behavioural similarities observed between the two species the interpretation of the underlying psychological motivation to ‘help’ differed significantly, with the former making no claims as regard the ant’s underlying motivation while the latter appealing to empathic concerns. While largely agreeing with Vasconcelos and colleagues’ critique, we further find the lack of alternative interpretations of the helping behaviours in both rats and ants limiting, since these would without doubt help to frame future studies in the field (e.g. see Heyes 2012 presentation of alternative interpretations of the Horner et al. 2011 study).

Finally, Massen et al. (2015) used a similar set-up with ravens to that employed by Skerry et al. (2011) with capuchin monkeys. A raven could deliver tokens to a partner, who in turn could exchange them for rewards. Results showed very few instances of prosocial behaviour and with no discernible pattern, leading the authors to conclude that results may have been due to the animals’ lack of understanding of the task (again highlighting the need for ‘knowledge probe’ trials to test for this).

Overall, studies looking at helping behaviour (defined as an act which, at the proximate level, is costly to the subject and beneficial only to the recipient) with non-human species have had difficulty in ruling out simpler explanations for the putatively helpful acts, such as a desire for social contact (Ben-Ami Bartal et al. 2011; Silberberg et al. 2013) or automatic actions triggered by prior training (Bräuer et al. 2013; Melis et al. 2011). Admittedly, detecting the underlying motivation for an action is not an easy task; however, careful variations in controlled settings may help. Indeed in this respect, studies with rats have been particularly useful (Silberberg et al. 2013).

### Food sharing

Food sharing has been defined as the transfer of a defendable food item from one food-motivated individual to another: for some authors this excludes theft (Feistner and McGrew 1986), while others have considered certain forms of theft (e.g. ‘tolerated theft’) and joint foraging as food sharing (Stevens and Gilby 2004). Food sharing is universal among humans and has received considerable attention as a model for the evolution of altruistic behaviour (Gurven 2004). It is also a relatively common form of prosocial behaviour among animals; however, the frequency and modality of food sharing vary considerably amongst species (see Stevens and Gilby 2004 for a review), and both the proximate and ultimate causes of these behaviours are still a matter of intense debate (Jaeggi et al. 2010a; Silk et al. 2013).

At the proximate level, positive reactions to approaches and tolerant sharing are likely to reflect prosociality, and negative reactions and forced transfers do not. In line with this reasoning, a number of different behavioural categories of food sharing have been identified in the literature, the main ones being: (a) unsolicited/spontaneous giving, i.e. active transfers (hand to hand; mouth to mouth; hand to mouth or vice versa) initiated by the owner in the absence of any form of request or begging by the recipient; (b) solicited sharing, i.e. active transfer of food by the owner following a request/begging gesture from the recipient; (c) tolerated theft/scrounging, i.e. a passive transfer in which the owner allows the recipient to take food directly from his/her hand/mouth; (d) co-feeding, i.e. a passive transfer in which the owner allows the other to take food that is in direct proximity and could be monopolized. Although some authors have suggested that the different types of food transfer may reflect differing levels of prosociality (Jaeggi et al. 2013), there is a need for caution, since some behavioural categories may simply apply more to a particular species than another due to their specific morphological and behavioural characteristics (e.g. passive transfer, where a chimpanzee takes food from the owners’ hand without the latter resisting, may not have an equivalent behaviour in no-hand species since food is either in the mouth or on the ground).

In terms of ultimate explanations, kin selection has been invoked to explain food sharing from parents (or related ‘helpers’) to infants (e.g. regurgitation in many gregariously living canid species Mech et al. 1999; Moehlman 1986, 1989; Geary 2000), and three explanations have been commonly proposed to explain food sharing among non-kin: (1) reciprocity (exchange of favours, food-for-sex/alliance etc.) (Trivers 1971, 2006), (2) harassment avoidance (or tolerated theft—Blurton Jones 1984; Stevens and Stephens 2002; Stevens 2004) and (3) a costly form of social signalling that may increase prestige (Zahavi 1975; Zahavi 1990) or show dominance (Rijksen 1978).

Most experimental studies of food sharing have used the same paradigm, that is, they provided individuals with clumped food to explore spontaneous food interactions (apes: Nissen and Crawford 1936; Jaeggi et al. 2010b; callitrichids: Feistner and Chamove 1986; Kasper et al. 2008; corvids: de Kort et al. 2006; Scheid et al. 2008), assessing the frequency of food transfer in relation to independently collected information on affiliative relationships, dominance, kinship etc. A number of studies have investigated food sharing in a dyadic rather than a group setting (de Waal 2000; Stevens 2004; Ostojić et al. 2013, 2014; Range et al. 2015), and more recently studies have included operant tasks (e.g. opening a door) to allow access to the partner and hence food sharing (Hare and Kwetuenda 2010; Tan and Hare 2013; Bullinger et al. 2013).

Most early studies tended to take into account only one potentially impacting variable at a time, but more recent studies have investigated multiple factors simultaneously, allowing a more complete picture to emerge (e.g. Eppley et al. 2013; Crick et al. 2013; Silk et al. 2013). Silk et al. (2013), for example, presented a small number (two or three) of divisible but monopolizable food items (i.e. frozen juice discs) to chimpanzees in a group setting. Observations were carried out taking into account both active and passive food transfers. Overall, transfers were related to the frequency and intensity of begging behaviour by the recipient, lending support to the harassment avoidance hypothesis (Stevens and Stephens 2002, Stevens 2004). However, both transfer types were directed more to close kin and reciprocating partners than to others when the frequency of begging was held constant.

A study by Jaeggi et al. (2010b) went one step further, focusing closely both on the different types of food transfers and the multiple factors which may affect these, while also comparing two different species (bonobos and chimpanzees). Surprisingly, considering results from other paradigms, in this more naturalistic food-transfer context chimpanzees appeared to be more prosocial than bonobos, and the authors suggested this may be due to the more relaxed dominance hierarchy that emerged in their chimpanzee populations. Interestingly, variables such as reciprocity and relatedness also affected the frequency of food transfer differently in the two species (Table 4).

**Table 4:** Food-sharing studies

Species	Group versus dyads	Task	Social effects	Effect of partner communication	Food type	Reciprocity	References
Chimpanzees	Dyads	Either in same or adjacent enclosures	None tested but authors report a possible effect of friendship	Begging behaviours analysed, but not in relations to transfer rates	Tokens transferred more frequently than food	NA	Nissen and Crawford (1936)
Bonobos	Dyads	Open a door to allow partner to share food	No effect of kinship, sex or in- versus out- group membership	No effect of solicitation	NA	NA	Hare and Kwetuenda (2010)
Bonobos	Dyads	Open a door to allow partner to share food	Shared with strangers as much (or more) as with familiar partners	No effect of solicitation	NA	NA	Tan and Hare (2013)
Chimpanzees	Group	Monopolizable food	More sharing towards kin	Solicitation increased food sharing	NA	Yes	Silk et al. (2013)
Chimpanzees	Group	Monopolizable food. Mixed male female group but only males were possessors	Males shared more with higher ranking females (regardless of perseverance) than low-mid ranking females No effect of affiliation	Perseverance in begging affected success rate of females obtaining low quality food from males but only for mid low rank females	High versus low quality	High quality food shared more with females who copulated with males (in the short term)	Crick et al. (2013)
Chimpanzees	Group (only females)	Monopolizable high quality food source	Close affiliative partners more likely to receive food with more perseverance. Low/no affiliative partners persevered less and received fewer food transfers. No effect of kinship and dominance	No effect of begging alone	NA	NA	Eppley et al. (2013)
Bonobos and chimpanzees	Group	Monopolizable food	Chimps shared more with kin and friends Bonobos shared up the hierarchy	NA	NA	Yes in chimps	Jaeggi et al. (2010b)
Bonobos and chimpanzees	Group	Monopolizable food	Chimps shared more with friends and kin	NA	NA	Bonobos (not chimps) showed short-term reciprocity (grooming/food)	Jaeggi et al. (2013)
Capuchins	Dyads	Food sharing across the mesh (food available to only one partner)	Only female-female dyads tested	NA	Food quality varied but no clear results	Yes	de Waal (2000)
Cotton-top tamarins	Group	Tasks varied the satiation level/and motivation for food items (more vs. less preferred)	Shares from adults to infants were studied	NA	More transfers when higher food quality and less satiation	NA	Feistner and Chamove (1986)
Marmosets	Dyads	Monopolizable food	More frequent from subordinates to dominants. No sex effect	NA	NA	More grooming if a food transfer occurred	Kasper et al. (2008)
Chimpanzees Bonobos Marmosets	Dyads	Open a door to allow partner to share food	No effect of partner or kinship	Solicitation decreased probability of sharing	NA	NA	Bullinger et al. (2013)
Chimpanzees Squirrel monkeys	Dyads	Monopolizable and sharable food	NA	Solicitation/harassment increased sharing	NA	NA	Stevens (2004)
Jackdaws	Group	Monopolizable food	Correlation between food sharing and allopreening	Solicitation increased food sharing	More sharing with high quality food	Yes	de Kort et al. (2006)
Jackdaws	Group	Active transfers and Stealing	Sharing occurred more with affiliative partners	No effect of solicitation	Shared more less preferred food	Unclear	von Bayern et al. (2007)
Rooks	Group	Active transfer and co-feeding	Active transfer more frequent down the hierarchy and from males than from females	No effect	NA	Co-feeding reciprocated. Active transfer not	Scheid et al. (2008)
Eurasian jays	Dyad	Active transfer	NA	Controlled for	Males fed females accounting for the latter’s food preference	NA	Ostojić et al. (2013, 2014)
Wolves and Dogs	Dyad	Monopolizable food	In dogs, only high-ranking subjects monopolized the food In wolves both high and low ranking subjects monopolized the food resource Wolves more tolerant than dogs	NA	More co-feeding in the meat than in the bone condition	NA	Range et al. (2015)

*NA* where a particular issues was not tested for

#### Critical issues in food-sharing studies

Studies on food sharing appear particularly useful in answering questions about the functional relevance of prosociality, hence investigating the multiple variables which may affect its emergence and maintenance. However, a potential limitation of the spontaneous sharing studies described above is the difficulty in teasing apart ‘coerced’ shares, i.e. when the possessor gives food up to avoid harassment by the beggar (although see Jaeggi et al. 2010b, for a detailed behavioural analyses of different types of sharing), from truly spontaneous shares (hence with an underlying prosocial motivation).

To address this problem, a novel approach has recently been adopted, allowing subjects to choose whether they would rather feed alone or in the presence of a known partner (Hare and Kwetuenda 2010). In the first study adopting this procedure, unrelated bonobos chose to interrupt their feeding and open the door to a conspecific, hence allowing the latter to also feed in the same room. In a more recent variant (Tan and Hare 2013), bonobos also showed a willingness to open the door and share food with a complete stranger. Unfortunately, because in both studies there was no control condition to assess whether bonobos would be equally likely to allow access to a known or unknown conspecific with no food present, it is not clear from these studies whether the bonobo’s preference for co-feeding was due to their motivation to *feed* together or, on a more basic level, by their high motivation to be together with another bonobo in the same room.

Using the same experimental approach, Bullinger et al. (2013) compared bonobos, chimpanzees and marmosets. However, importantly, the authors included the control condition lacking in the Tan and Hare study, namely a condition where subjects could choose to open the door for a partner without food being present in the room. Hence in this respect it taps into the prosocial motivation of the subject. In contrast to the other study, chimpanzees, bonobos and marmosets did not voluntarily co-feed, although they did choose to give access to a conspecific if no food was present. However, in requiring the animal to make a choice to open a door for a partner while already having gained access to the valued resource, a potentially onerous inhibitory control element is included in the task. A difficulty in inhibiting ones’ own feeding response may therefore mask the potential prosocial motivation. Perhaps a better option, so far adopted only with chimpanzees (Bullinger et al. 2011), is to require subjects to make the choice before accessing the food. Indeed in the study by Bullinger et al. (2011), subjects preferred accessing an enclosure containing an apparatus delivering food only to them than an enclosure with a cooperative apparatus, which allowed both it and a partner to obtain food.

Overall, food-sharing studies, in particular the experimental approach involving dyads, have suffered from the same problems as ‘helping studies’, i.e. detecting the underlying motivation of animals. Indeed once the desire for social contact per se was controlled for, food sharing (Bullinger et al. 2013) like helping (Silberberg et al. 2013) was no longer as evident. Similarly, in a group setting, the difficulty of assessing the underlying motivation remains, with ‘harassment avoidance’ rather than ‘prosociality’ being the more likely alternative (Stevens 2004).

A second concern is that studies on food sharing in captivity are not always grounded in the species’ natural ecology. Primate species in the wild differ quite significantly in their prevalence of food-sharing behaviour, although this is mostly directed at their infants (Rapaport and Brown 2008). Furthermore, food sharing appears to be more widespread in other taxa (e.g. corvids and canids, de Kort et al. 2006; Emery 2004; Creel and Creel 2001; Packard 2003). Considering the natural ecology of the various tested species, it is perhaps not surprising that experimental studies of food sharing in captivity in corvids (see section below) have been overall more successful in teasing apart the nuances of this behaviour than those in primates (Thornton and McAuliffe 2015).

Nevertheless, studies using food-sharing paradigms are promising especially since presentation in a group environment, coupled with more sophisticated statistical treatment of multiple variables, allows for a better understanding of the factors affecting the emergence and maintenance of food sharing. Furthermore, the relative simplicity of the paradigm potentially allows for wider across-species comparisons.

#### Food-sharing studies in non-primates

Compared to other prosocial paradigms (PCT and helping), food-sharing studies have been extended more often to include non-primate species, although, thus far, the focus has been mainly on different corvid species (de Kort et al. 2006; von Bayern et al. 2007; Scheid et al. 2008; Ostojić et al. 2013, 2014).

As in similar work with primates, most studies have presented food-sharing paradigms in a group setting. For example, de Kort et al. (2006) investigated juvenile jackdaws’ food-sharing behaviour in a group setting and found that jackdaws actively food share (beak-to-beak) with a number of individuals regardless of sex, dominance relationship and kinship, but that most food transfers were ‘solicited’, lending support to the ‘harassment avoidance’ hypothesis. However, (independent of begging), most transfers involved a more, rather than less, preferred food and were positively related to allopreening, suggesting that food transfer may have a role in the establishment and maintenance of social bonds (Emery 2004). A reciprocity effect also emerged, with food transfers being more frequent towards partners from whom food had been received in the past (although see von Bayern et al. 2007).

Importantly, another corvid study carried out in a group setting, this time on rooks, also pointed to potential differences in the functional relevance of co-feeding (allowing another to approach and feed together) and active transfer (beak-to-beak), in that the latter was related to dominance and sex, while the former was reciprocated and associated with pair-bond formation (Scheid et al. 2008). Hence, food-sharing studies in a group context in corvids have been particularly useful in teasing apart the potential functions not just of food sharing, but of the different types of food sharing.

Two studies, also on corvids, are particularly interesting because they aimed at understanding the underlying motivation for food sharing (Ostojić et al. 2013, 2014). Using an innovative approach, researchers tested whether male Eurasian jays would take a female’s desire for one food type over another into account when choosing to share food with her. Results showed that if male jays were allowed to witness their partner reaching satiation on one food type (e.g. mealworms), they would systematically choose to share an alternative food with her (e.g. wax moth larvae), leading the authors to suggest that jays are capable of taking the ‘desire-state’ of their partner into account (Ostojić et al. 2013). However, it is perhaps important to note that the main result is not overly robust and is based on a one-tailed analysis (p. 4124). In a subsequent study, the researchers also showed that if the males’ own desire was in conflict with the females’, they still took their partners’ state into account, although they struggled to do so (Ostojić et al. 2014).

Finally, in a recent study wolves and dogs were compared in their tolerance for food sharing in a dyadic context (Range et al. 2015). The aim of this study was to start exploring the effect of social dynamics on food-sharing behaviours, and potential differences between the two species. What emerged was that in wolves, both dominant and subordinate members of the dyads monopolized the food and showed agonistic behaviours to a similar extent, whereas in dogs these behaviours occurred predominantly in high-ranking animals. Results showed that dominance relationships affected the two species differently in a feeding context, suggesting this factor may be an important variable to consider in studies on prosociality in different species.

## Conclusions

As is abundantly clear from the present review, most experimental studies on prosociality and altruism in non-human species have focused on primates, with only a few studies exploring these topics in other species. As argued in the introduction, there is, however, a need to broaden the range of species to better understand the social and ecological factors which may influence the evolution of prosocial tendencies. Indeed, within the primate order at least, recent efforts have been made to widen the comparison across species with different social and ecological environments (e.g. Bullinger et al. 2013; Burkart et al. 2014).

The challenge we face in attempting to include non-primate species in these comparisons is to develop experimental paradigms which effectively test animals’ prosocial/altruistic tendencies without making overly complex cognitive demands and which can be adopted equally efficiently by species with different morphological characteristics.

In light of this, the food-sharing paradigm appears to be particularly suitable since: (a) it is a spontaneous, observable behaviour with high ecological validity requiring no prior training; (b) it allows for experimental manipulations mirroring those found in the natural environment, for example via manipulation of food abundance, distribution and quality; (c) it keeps the group environment intact allowing individuals to spontaneously choose from the whole host of potential ‘partners’ hence avoiding potentially artificial effects due to ‘unnatural/unlikely’ dyadic combinations; (d) but it can also be presented in a dyadic format to evaluate the effect of specific variables one at a time; and (e) with a careful definition of the different forms of food transfer (solicited vs. unsolicited, forced vs. relaxed transfer, passive vs. active, co-feeding at different proximities etc.), it may allow more subtle and comparable analyses of the prosocial tendencies across species. However, food-sharing paradigms, like the other paradigms presented here, need to be carefully designed to tease apart the underlying motivational and cognitive processes (e.g. Ostojić et al. 2013, 2014). Furthermore, food sharing involves a cost to the actor, and this may mask the animals’ willingness to benefit others when this is not associated with high costs (or example in the 1/0 vs. 1/1 PCT set-up). In this respect it may not be the most sensitive way to assess prosocial behaviours. Finally, as argued above, active sharing is rare and especially in spontaneous food-sharing tasks/contexts, differences in the morphology of the species may render such a behaviour difficult, if not impossible, to detect. It is therefore important to take into account the ecological validity of the behaviour for the species studied.

A task that does measure active sharing and allows manipulation of the immediate cost to the actor, is the PCT paradigm which, has now been extended to studies with rats, dogs and birds. However, considering the additional cognitive demands (especially of the token test), it is particularly important to test the animal’s understanding of the task, since in some of the primate studies where knowledge tests were included, results actually shed doubts on the subject’s understanding (Brosnan et al. 2009).

A possible way forward would be to adapt the current PCT paradigms to reduce cognitive demands. Indeed both Burkart and Rueth’s (2013) study with children, and House et al.’s (2014) with chimpanzees, found that subjects made fewer prosocial choices the more complex the task, i.e. the greater number of options present in the experimental paradigm. Hence converging evidence seems to indicate the need to simplify tasks to test a species’ ‘prosocial tendency’ whilst avoiding potentially confounding variables, such as the species’ capacity to cope with the cognitive demands of the test (see also Seed et al. 2012). In this respect the version of the bar-pulling used in studies with dogs (Quervel-Chaumette et al. 2015) and marmosets (Burkart et al. 2007) may be promising. The subjects incur an immediate cost (0/0 vs. 0/1, i.e. they pull for others but obtain nothing themselves) which may arguably be a better measure of an underlying ‘other-regarding’ motivation, and having to follow a single food item constitutes a cognitively and ‘attentionally’ simpler version compared to the standard 1/0 versus 1/1 paradigm. Based on the currently available evidence with this version, extinction-type paradigms, where a response can either be performed or not, have successfully been used not just with dogs, but also with rats (in a generalized reciprocity framework not discussed here: Rutte and Taborsky 2007, 2008; Schneeberger et al. 2012) and a wide range of primate species (e.g. Burkart et al. 2013, 2014) suggesting it may be a fruitful avenue for cross-species comparisons, when coupled with the necessary control conditions to insure the animal’s understanding of the task. Along the same lines, in that it also reduces the cognitive demand of the task, the recent PCT version using a T-maze paradigm (Hernandez-Lallement et al. 2015), may also provide a good set-up for comparative analyses across species.

An important issue raised by Thornton and McAuliffe (2015) is to what extent such laboratory paradigms truly reflect the food-sharing behaviours observed by the species in the wild. Perhaps a start in this direction would be to at least use both PCT and food-sharing paradigms in captivity to start investigating whether the same pattern emerges using both methods.

Moreover, further attention is needed to the social dynamics of each species and the consequent pairing of donor and recipient; an issue which clearly emerged in food-sharing studies with rooks (Scheid et al. 2008), but which has not always been taken into consideration in PCT studies. Indeed, an interesting way forward may be the application of the PCT paradigm in a group setting, where animals have the freedom to choose which partner to provision (Burkart and van Schaik 2013; Burkart et al. 2014; House et al. 2014).

Overall, more recent studies have recognized the benefits of a wider comparative approach to the study of prosociality, testing multiple species with the same task (e.g. Amici et al. 2014; Burkart and van Schaik 2013; Burkart et al. 2014); however, these have still focused predominantly on primates. Yet, to answer questions as regards the evolutionary pressures shaping the occurrence of prosocial tendencies, a wider perspective is needed. Experimental paradigms developed to test primates, may be successfully employed with other species, in particular if efforts are made to simplify tasks and ascertain the animals’ understanding of test contingencies.
